# The relationship between peer support and sleep quality among Chinese college students: the mediating role of physical exercise atmosphere and the moderating effect of eHealth literacy

**DOI:** 10.3389/fpsyg.2024.1422026

**Published:** 2024-07-31

**Authors:** Jin Li

**Affiliations:** Department of Physical Education, Beijing Union University, Beijing, China

**Keywords:** college students, peer support, physical exercise atmosphere, eHealth literacy, sleep quality

## Abstract

**Background:**

Poor sleep quality has emerged as a prevalent health issue among college students. This study aims to explore the mechanism of sleep quality among college students by constructing a moderated mediation model.

**Methods:**

The Peer Support Scale, Physical Exercise Atmosphere Scale, eHealth Literacy Scale and Pittsburgh Sleep Quality Index were used to conduct a survey and analysis on 1,085 questionnaires, which were distributed among students from four universities in the northwest, northeast and central regions of China.

**Results:**

(1) A significant pairwise correlation exists between peer support, physical exercise atmosphere, eHealth literacy and sleep quality (*P* < 0.001); (2) Physical exercise atmosphere plays a mediating role between peer support and sleep quality, with a mediating effect accounting for 28.08%; (3) eHealth literacy can significantly moderate the strength of the relationships between peer support and exercise atmosphere, between peer support and sleep quality, and between physical exercise atmosphere and sleep quality. This study revealed the relationship between peer support and sleep quality among college students and its influencing mechanism, and provided theoretical and practical basis for improving college students' sleep quality from the perspectives of peer support, physical exercise atmosphere, and eHealth literacy.

## 1 Introduction

Sleep is a fundamental physiological activity in the human body, playing a crucial role in ensuring the normal functioning of individuals' immune system (Bryant et al., 2004), metabolic system (Xie et al., 2013), and brain (Hanlon and Van Cauter, 2011). In recent years, with the increasing complexity of the social environment, poor sleep quality among college students has become increasingly prominent. Survey findings indicated that as many as 60% of college students suffered from sleep-related problems, including limited sleep duration and challenges in initiating sleep (Foulkes et al., 2019). Sleep is an important factor that directly affects individuals' physical and mental health. Studies indicated that a decrease in sleep quality not only leads to cognitive impairment (Roberts et al., 2008) and emotional disorders in college students (Khazaie and Chehri, 2017), but also induces unhealthy behaviors such as smoking and alcohol abuse (Tao et al., 2017), and even increases the risk of suicidal thoughts (Castelnuovo et al., 2021). Therefore, it is of great practical significance to identify the factors that influence the sleep quality of college students and thoroughly explore their formation mechanisms. The ecosystem theory posits that (Bronfenbrenner, 1979), beyond the family, the school environment constitutes a significant social factor impacting students' attitudes, motivations, and behaviors (Mao et al., 2019). College students, engaged in frequent peer social interactions, often prefer to seek advice or support from their peers to address their non-adaptive behaviors (Wu and Chen, 2016). Therefore, peer support, as a crucial element within the school environment, can influence college students not only through various forms of support such as emotional, environmental, and instrumental support, but also subtly shape their health consciousness and behaviors by fostering a strong culture of physical activity (Fitzgerald et al., 2012). Amid the swift advancement of the Internet, eHealth literacy emerges as a crucial variable potentially influencing individual emotions, health behaviors, and sleep quality. In view of this, this study took college students as research subjects, examined key variables including peer support, exercise ambiance, and e-health literacy, and explored the internal mechanisms that affect the sleep quality of college students, in order to provide reference for improving their sleep quality.

### 1.1 Peer support and college students' sleep quality

**Figure 1:**
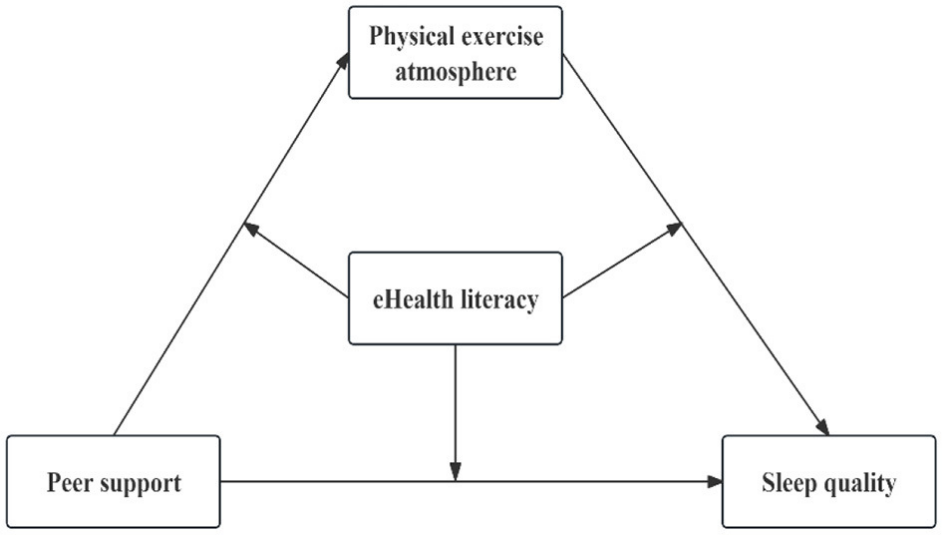
Hypothetical theory model.

Peer support refers to the support from individuals of similar age, living environments, experiences, culture, social statuses, or who share the same language, exchange information, ideas, emotions, or behavioral skills (Dennis, 2003). Peer support can offer emotional, informational, and instrumental support (Solomon, 2004) to adolescents, establishing a system of giving and receiving assistance founded on respect, sharing, and mutual aid. Studies indicated a strong similarity in healthy behaviors among peers (Hamm, 2000). Positive peer relationships can furnish individuals with support and help beyond their own capabilities (Li et al., 2019), fulfilling social needs, providing positive emotional experiences (de Alcantara et al., 2019), deterring problematic behaviors, and thereby positively affecting individual sleep quality. According to the social support buffer model, social support received from immediate social connections serves as a crucial protective factor when individuals are exposed to prolonged high-risk environments (Cohen and Wills, 1985). As a significant form of social support for adolescents (Zhou et al., 2019), peer support can mitigate potential stressful events and minimize external issues as much as possible (Lansford et al., 2003), thereby alleviating sleep quality problems like sleep disorders and insomnia. Furthermore, according to the theory of sleep disturbance process, the negative psychological emotions induced by excessive emotional arousal can disrupt the normal sleep cycle, leading to deteriorated sleep quality (Lundh and Broman, 2000); however, perceiving high-quality peer support could mitigate the impact of adverse pre-sleep emotions on the sleep cycle, thereby enhancing sleep quality. Based on the theoretical and empirical studies mentioned above, this study proposes the Hypothesis 1: Peer support can significantly predict college students' sleep quality (as shown in Figure 1).

### 1.2 The moderating role of physical exercise atmosphere

Physical exercise atmosphere refers to the supportive atmosphere created by social and natural environments that encourage individual participation in sports activities, playing a crucial mediating role between psychological traits and exercise behaviors (Pan et al., 2010). As a vital component of the external physical activity environment, physical exercise atmosphere is significantly correlated with interpersonal relationships (Liang et al., 2018); moreover, interpersonal relationships can positively influence physical exercise atmosphere (Cheng, 2022). According to Behavioral Learning Theory, adolescents in the stage of adapting to social development are more inclined to form specific psychological traits and behaviors under peer support (Liao et al., 2016). This indicates that friendships among peers play a crucial role in the development of social interpersonal relationships by motivating individuals to embrace behavior patterns that align with their social surroundings. This alignment, in turn, fosters a more positive and harmonious interpersonal atmosphere (Zhu and Qiu, 2006). Expanding individuals' social network leads to positive exchanges of trust and emotional communication (Yu and Zhu, 2019), advancing social network connections, enhancing emotional connections among groups and societal identification, and thus encouraging peer groups to actively engage in physical and artistic activities together (Liu, 2016; Costigan et al., 2019). Thus, peer support is likely to positively influence the physical activity environment. Furthermore, based on Emotional Memory theory, emotions evoked by the social environment (such as joy, anxiety, fear, anger) become cognitive symbols in memory, reshaping the cognitive system and influencing future behaviors (Bower, 1981). The physical exercise atmosphere reflects the social environment of college students, and exerts a significant radiating effect on individual health behaviors (Lepore and Greenberg, 2002). That is, when a positive physical exercise atmosphere provides an encouraging environment or setting for adolescents to exercise, it helps to elicit positive emotional responses, enhance the ability to adapt to the environment, cultivate healthy living habits (Wang, 2014), and subsequently improve the possibility of enhancing individual sleep quality. Accordingly, this study proposed Hypothesis 2: Peer support can affect college students' sleep quality through the mediating role of the physical exercise atmosphere (as shown in Figure 1).

### 1.3 The mediating effect of eHealth literacy

eHealth literacy refers to the ability of individuals to seek, comprehend, assess, and apply health information from electronic sources to address personal health issues. Compared to health literacy, eHealth literacy emphasizes more on the ability to acquire and apply health knowledge through electronic media (Norman and Skinner, 2006a). A large number of studies showed that eHealth literacy is significantly positively correlated with individual health-related behaviors (Suka et al., 2015; Kim et al., 2023). The Knowledge—Attitude/Belief—Practice model suggests that when people acquire knowledge and engage in positive thinking about it, a strong positive cognition is generated, which in turn drives knowledge to become a belief and enables individuals to adopt a positive attitude to change behaviors (Pärna et al., 2005). From this perspective, individuals with high eHealth literacy levels may possess strong health beliefs, and they usually more actively search for relevant health information on Internet platforms (Britt and Hatten, 2013), and communicate and share based on relevant information (Segal et al., 2012) to gain more social support (Ha et al., 2023). High eHealth literacy not only fosters a sense of understanding and respect from others, boosting subjective well-being and sense of belonging (Zhao and Wang, 2016); it also enhances individual lifestyle and health behavior (Liu and Fu, 2024), encourages activities like physical exercise, leisure and entertainment, triggers peer effects, enhances group identity (Cheng and Dong, 2018), and promotes stable and positive emotional effects, thereby relieving stress and improving sleep quality. Furthermore, individuals with high eHealth literacy levels often have strong health awareness (Cho et al., 2014) which can be internalized as an intrinsic motivation to actively participate in exercise and pay attention to the value of sleep in practice (Miao et al., 2020), thereby helping individuals form healthy exercise and lifestyle habits. In view of this, this study proposes Hypothesis 3: eHealth literacy can significantly moderate the effects of peer support on sleep quality, physical exercise atmosphere on sleep quality, and peer support on exercise atmosphere (as shown in Figure 1).

In summary, this study constructed a moderated mediation model by integrating multiple theories and previous research. This model was used to explore the relationship between peer support and college students' sleep quality, with a specific focus on the mediating and moderating effects of physical exercise atmosphere and eHealth literacy, in order to further clarify the relationship between peer support and sleep quality. The hypothetical theory model is illustrated in Figure 1.

## 2 Research design

### 2.1 Data and participants

Using the convenience sampling method, a questionnaire survey was conducted on students from four universities located in the northwest, northeast, and central regions of China, totaling 1200 respondents, through WJX online survey platform. By filtering out questionnaires with excessively short completion times and missing responses, a total of 1,085 valid questionnaires were obtained, with a valid response rate of 90.42%. Among the respondents, there were 656 males (60.46%) and 429 females (39.54%); in terms of age, 98 respondents were 18 and below (9.03%), 263 were 19 years old (24.24%), 336 were 20 years old (30.97%), 196 were 21 years old (18.06%), 111 were 22 years old (10.23%), and 81 were 23 years old and above (7.47%). In terms of geographical distribution, 304 college students came from rural areas (accounting for 28.02%), while 781 college students came from urban areas (accounting for 71.98%). Regarding their field of study, 256 were majored in humanities (23.59%) and 829 were majored in science and engineering (76.41%).

Before the survey, the subjects were clearly informed of the purpose of the study and the voluntary nature of participation in the questionnaire, and the anonymity and confidentiality of the data collection were emphasized, informing them that the data collected would be used only for scientific research and that no information would be disclosed. Data collection was carried out on the basis of obtaining the subjects' permission and signing an informed consent form. Finally, the studies involving humans were approved by the Division of Science and Technology, Beijing Union University, and they were conducted in accordance with local legislation and institutional requirements.

### 2.2 Research tools

#### 2.2.1 Peer support scale

This study adopted the revised version of the Perceived Social Support Scale which was initially developed by Zimet et al. (1988), and later modified by Huang et al. (1996). The scale consists of three subdimensions: family support, peer support, and other forms of support. In this study, the Peer Support Scale, which includes four items, was chosen to measure the level of peer support among college students. It adopts a 5-point Likert scale, with scores ranging from 1 (totally disagree) to 5 (totally agree). A higher score indicates greater perceived peer support by the individual. Confirmatory factor analysis showed that X^2^/df = 3.626, *GFI* = 0.998, *AGFI* = 0.983, *NFI* = 0.999, *RFI* = 0.994, *IFI* = 0.999, *TLI* = 0.996, *CFI* = 0.999, *RMSEA* = 0.049. The good fit indices indicated that the questionnaire has good validity. The Cronbach's alpha coefficient for the peer support scale was measured to be 0.928, indicating that the questionnaire has good reliability.

#### 2.2.2 Physical exercise atmosphere

This study adopted the Physical Exercise Atmosphere Scale revised by Chen (2010). The physical exercise atmosphere was measured from three dimensions: group physical exercise atmosphere, the reception of exercise media information, and the physical exercise behaviors of surrounding people. The scale consists of 12 items, using a 5-point scoring system where 1 means “strongly disagree” and 5 means “strongly agree”. A higher score indicates a stronger perceived physical exercise atmosphere. Confirmatory factor analysis showed that *X*^2^*/df* = *7.559, GFI* = 0.945, *AGFI* = 0.912, *NFI* = 0.972, *RFI* = 0.963, *IFI* = 0.976, *TLI* = 0.967, *CFI* = 0.976, *RMSEA* = 0.078. The good fit indices indicated that the questionnaire has relatively good validity. The calculated Cronbach's α coefficient of the physical exercise atmosphere scale was 0.941, indicating that the scale has good reliability.

#### 2.2.3 eHealth literacy scale

This study adopted the eHealth Literacy Scale which was developed by Norman and Skinner (2006b) in 2006, and later translated into Chinese by Guo et al. (2013). The scale consists of 8 items, using a 5-point scoring system where 1 means “strongly disagree” and 5 means “strongly agree”. The total score for each respondent is the sum of item scores, ranging from 8 to 40. Scores above 32 indicate “Qualified”, and higher scores indicate higher eHealth literacy levels. Confirmatory factor analysis showed that *X*^2^*/df* = 7.272, *GFI* = 0.977, *AGFI* = 0.937, *NFI* = 0.990, *RFI* = 0.979, *IFI* = 0.992, *TLI* = 0.982, *CFI* = 0.992, *RMSEA* = 0.076. The good fit indices indicated that the questionnaire has relatively good validity. The calculated Cronbach's α coefficient of the eHealth literacy scale was 0.946, indicating that the questionnaire has good reliability.

#### 2.2.4 Pittsburgh sleep quality index

Sleep quality was measured by using the Pittsburgh Sleep Quality Index compiled by Buysse et al. (1989). The scale consists of 18 items, including seven factors: sleep quality, sleep latency, sleep duration, sleep efficiency, sleep disturbance, and daytime dysfunction. The scale employs a 4-point scoring method, with scores ranging from 0 to 3 based on the degree of sleep quality. The total score is obtained by summing all the items, with higher scores indicating poorer sleep quality. In this study, the Cronbach's α coefficient for the scale was 0.830.

### 2.3 Research procedure

In this study, all respondents read the informed consent form and agreed to voluntarily participate in the survey. Secondly, all respondents completed the questionnaire in a quiet indoor environment. Finally, data for this study were selected by removing invalid questionnaires that violated response rules, had insufficient completion time, or contained missing responses.

Data analysis in this study was performed using SPSS 26.0. Descriptive statistics were used to describe the demographic information of the participants. Pearson correlation analysis was used to assess the relationships between variables. The moderating mediation model was constructed using the PROCESS3.3 macro program, which utilizes bootstrap resampling. The 95% confidence interval (CI) was calculated based on 5,000 bootstrap samples to estimate the mediating and moderating effects. Results were considered statistically significant if the confidence interval did not include zero.

## 3 Results and analysis

### 3.1 Common method deviation test

Harman's single factor test was adopted. All subjects related to the four variables (peer support, physical exercise atmosphere, eHealth literacy, and sleep quality) were included in the exploratory factor analysis. Nine factors with eigenvalues >1 were calculated, which explained 72.811% of the variation. The explained variation of the first factor was 17.007%, which was less than the critical value of 40%. This indicated that there was no obvious common method bias in the survey process.

### 3.2 The mean, standard deviation, and correlations of different variables

Descriptive statistics and correlation analysis results indicated that (Table 1), peer support, physical exercise atmosphere, eHealth literacy and sleep quality have significant correlation with each other (*P* < 0.01). Peer support is significantly positively correlated with physical exercise atmosphere (*r* = 0.241, *P* < 0.01) and eHealth literacy (*r* = 0.278, *P* < 0.001), while physical exercise atmosphere is significantly positively correlated with eHealth literacy (*r* = 0.595, *P* < 0.001); Sleep quality is significantly negatively correlated with peer support (*r* = −0.223, *P* < 0.01), physical exercise atmosphere (*r* = −0.205, *P* < 0.01), and eHealth literacy (r = −0.201, *P* < 0.01). To conclude, all variable correlations achieved statistical significance, preliminarily suggesting that peer support, physical exercise atmosphere, and eHealth literacy could enhance sleep quality. This suggests that further statistical analysis can be conducted.

**Table 1:** Standard deviation, mean and correlation matrix of each variable.

**Variable**	**M ±SD**	**Peer support**	**Physical exercise atmosphere**	**eHealth literacy**	**Sleep quality**
Peer support	14.595 ± 3.036	1			
Physical exercise atmosphere	36.039 ± 10.143	0.241^**^	1		
eHealth literacy	28.862 ± 5.657	0.278^***^	0.595^***^	1	
Sleep quality	6.096 ± 3.220	−0.223^**^	−0.205^**^	−0.201^**^	1

^*^P < 0.05, ^**^P < 0.01, ^***^P < 0.001.

### 3.3 Mediating effect analysis

Mediation effect was tested for the relationship among peer support, physical exercise atmosphere and sleep quality by using the PROCESS plug-in compiled by Hayes and using model 4 under controlled conditions (including gender, age, place of origin, and major). The results (as shown in Table 2) showed that, without mediating variables, peer support exerted a significantly negative impact on sleep quality (β = −0.214, *t*= −7.155, *P* < 0.001), indicating that enhanced peer support can effectively improve sleep quality, thus Hypothesis 1 is supported. Peer support exerted a significant positive impact on physical exercise atmosphere (β = 0.316, *t* = 11.021, *P* < 0.001), and the exercise atmosphere exerted a significant negative impact on sleep quality (β = −0.190, *t* = −6,088, *P* < 0.001). This indicates that peer support can improve physical exercise atmosphere, while physical exercise atmosphere can improve sleep quality. This preliminarily verified the mediating effect of physical exercise atmosphere on the relationship between peer support and sleep quality among college students.

**Table 2:** Analysis of the mediating effect of irrational procrastination behavior.

**Variable**	**Sleep quality**					**Physical exercise atmosphere**					**Sleep quality**				
	**β**	**t**	***P***	**LLCI**	**ULCI**	**β**	**t**	***P***	**LLCI**	**ULCI**	**β**	**t**	***P***	**LLCI**	**ULCI**
Gender	0.14	4.243	0	0.075	0.205	−0.194	−6.143	0	−0.257	−0.132	0.103	3.121	0.002	0.038	0.168
Age	−0.02	−0.678	0.498	−0.079	0.039	0.019	0.673	0.501	−0.037	0.076	−0.017	−0.564	0.573	−0.074	0.041
Place of origin	−0.018	−0.609	0.543	−0.077	0.04	−0.034	−1.181	0.238	−0.09	0.022	−0.025	−0.838	0.403	−0.082	0.033
Major	−0.004	−0.126	0.899	−0.068	0.06	0.02	0.631	0.528	−0.041	0.081	0	−0.012	0.991	−0.063	0.062
Peer support	−0.214	−7.155	0	−0.272	−0.155	0.316	11.021	0	0.26	0.372	−0.154	−4.96	0	−0.215	−0.093
Physical exercise atmosphere											−0.19	−6.088	0	−0.251	−0.129
R	0.246					0.368					0.303				
R2	0.06					0.135					0.092				
F	13.881					33.719					18.131				

All variables in the model are standardized data. This is also applicable to the table below.

In addition, Bootstrap analysis was used to conduct the mediation path test. The results showed that (see Table 3), in terms of direct effects, the lower limit of the 95% confidence interval of peer support on sleep quality was −0.2145, and the upper limit was −0.0929. This interval did not contain 0, indicating that health belief can directly and negatively influence sleep quality. The effect value was −0.1537, and direct effects accounted for 71.92%. For the mediating effect of physical exercise atmosphere, the lower limit of the 95% confidence interval was −0.0834, and the upper limit was −0.0388. This interval did not contain 0, indicating that physical exercise atmosphere played a mediating role between peer support and sleep quality. The effect value was −0.0601, and mediating effects accounted for 28.08%. The establishment of the mediating effect demonstrated that peer support not only directly and positively influence the sleep quality of college students, but also indirectly influence it through physical exercise atmosphere. Therefore, *Hypothesis 2* is supported.

**Table 3:** Bootstrap analysis for the significance test of the mediating effect.

**Effect paths**	**Effect size**	**Boot**	**Boot CI**	**Boot CI**	**Relative effect ratio/%**
		**Standard error**	**Lower limit**	**Upper limit**	
Peer support → Sleep quality	−0.1537	0.031	−0.2145	−0.0929	71.92%
Peer support → Physical exercise atmosphere → Sleep quality	−0.0601	0.0116	−0.0834	−0.0388	28.08%
Total effect	−0.2137	0.0299	−0.2724	−0.1551	100%

### 3.4 Moderated mediation model test

**Figure 2:**
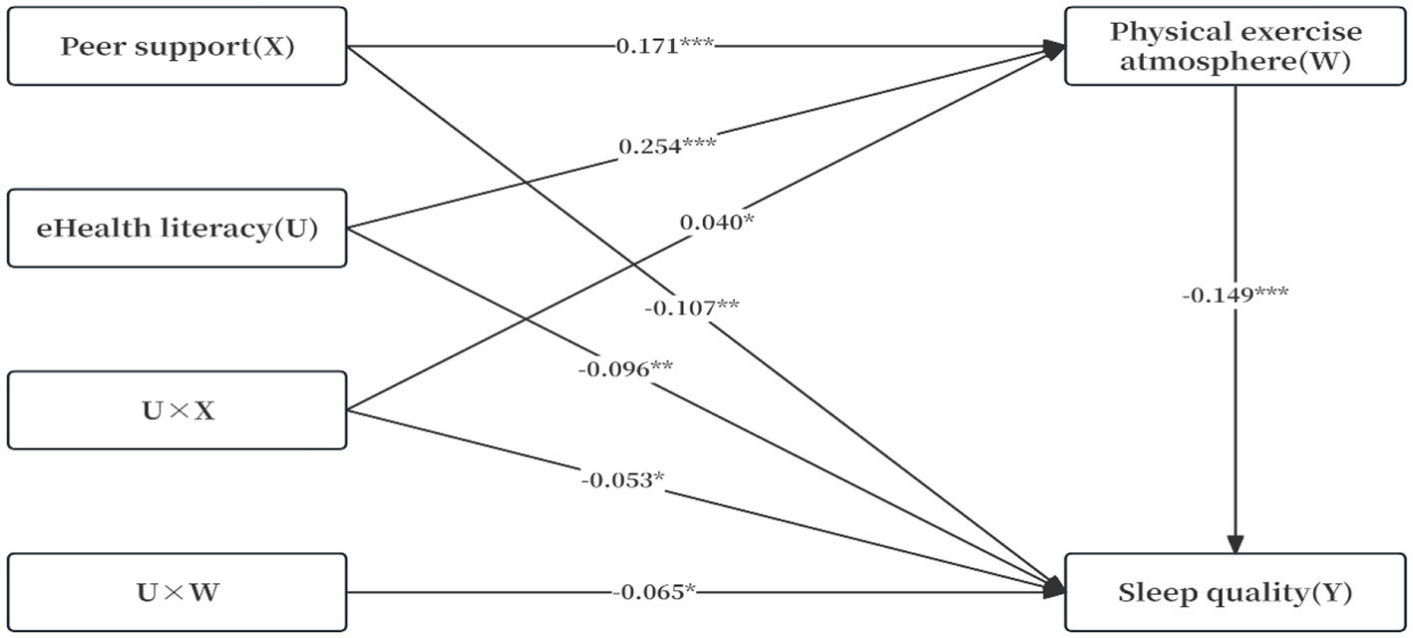
Moderated mediation model. ^*^P <0.05, ^**^P <0.01, ^***^P <0.001.

In terms of moderating effects (Table 4, Figure 2), the PROCESS plug-in was used and Model 59 was selected to test the moderated mediation model under controlled conditions (including gender, age, place of origin, and major). After incorporating eHealth literacy into the mediation model, the interaction between peer support and eHealth literacy exerted a significant positive impact on physical exercise atmosphere (β = 0.040, *t* = 2.223, *P* < 0.05), but a significant negative impact on sleep quality (β = −0.053, *t* = −2.296, *P* < 0.05). The interaction between physical exercise atmosphere and eHealth literacy exerted a significantly negative impact on sleep quality (β = −0.065, *t* = 2.466, *P* < 0.05). Therefore, Hypothesis *3* was validated.

**Table 4:** Moderated mediation model test.

**Variable**	**Physical exercise atmosphere**					**Sleep quality**				
	**β**	**t**	***P***	**LLCI**	**ULCI**	**β**	**t**	***P***	**LLCI**	**ULCI**
Gender	−0.185	−5.977	0.000	−0.246	−0.124	0.095	2.904	0.004	0.031	0.159
Age	0.001	0.048	0.962	−0.054	0.057	−0.010	−0.336	0.737	−0.067	0.048
Place of origin	−0.029	−1.040	0.299	−0.084	0.026	−0.028	−0.950	0.342	−0.085	0.029
Major	0.016	0.510	0.610	−0.044	0.075	−0.004	−0.117	0.907	−0.066	0.058
Peer support	0.171	5.010	0.000	0.104	0.238	−0.107	−2.968	0.003	−0.178	−0.036
eHealth literacy	0.254	7.439	0.000	0.187	0.321	−0.096	−2.624	0.009	−0.168	−0.024
Peer support × Health literacy	0.040	2.223	0.026	0.005	0.076	−0.053	−2.296	0.020	−0.098	−0.008
Physical exercise atmosphere						−0.149	−4.604	0.000	−0.213	−0.086
Physical exercise atmosphere × eHealth literacy						−0.065	−2.466	0.014	−0.117	−0.013
R	0.424					0.342				
R2	0.180					0.117				
F	33.741					15.830				

**Figure 3:**
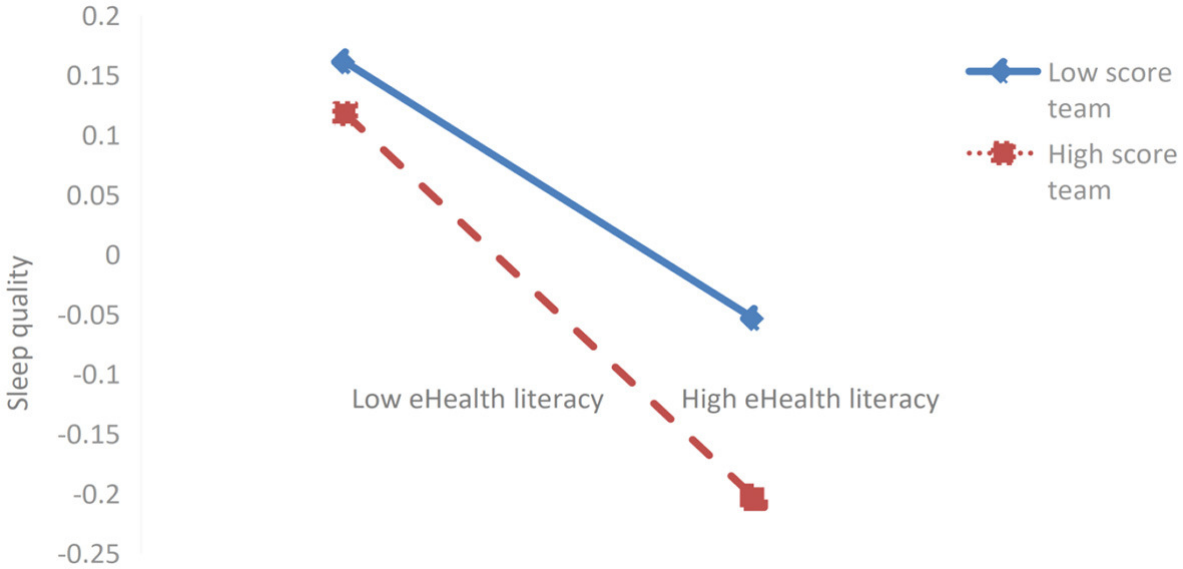
The mediating effect of eHealth literacy on the relationship between peer support and sleep quality.

**Figure 4:**
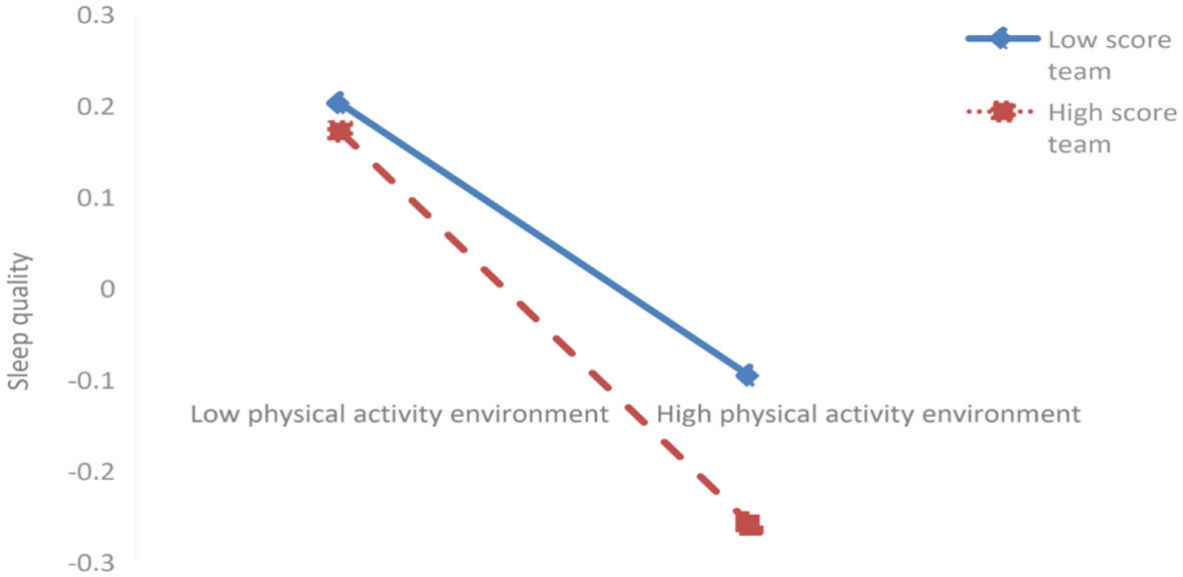
The moderating effect of eHealth literacy on the relationship between physical activity environment and sleep quality.

Further simple slope analysis revealed (as shown in Figures 3, 4) that for students with lower scores in eHealth literacy (*M*-1SD), peer support (*simple slope* = −0.054, *t* = −1.306, *P* > 0.05) and physical exercise atmosphere (*simple slope* = −0.084, *t* = −1.819, *P* > 0.05) exerted no significant negative impact on sleep quality. For students with higher scores on eHealth literacy (*M* + 1SD), peer support (*simple slope* = −0.160, *t* = −3.620, *P* < 0.001) and physical exercise atmosphere (*simple slope* = −2.15, *t* = −5.774, *P* < 0.001) exerted a significantly negative impact on sleep quality. This indicates that eHealth literacy can significantly mediate the intensity of the impact of peer support and physical exercise atmosphere on sleep quality. Specifically, when the score on eHealth literacy increased, the negative impact of peer support and physical exercise atmosphere on sleep quality showed an upward trend (sleep quality improved); when the score on eHealth literacy decreased, the negative impact of peer support and exercise atmosphere on sleep quality showed a downward trend (sleep quality deteriorated).

**Figure 5:**
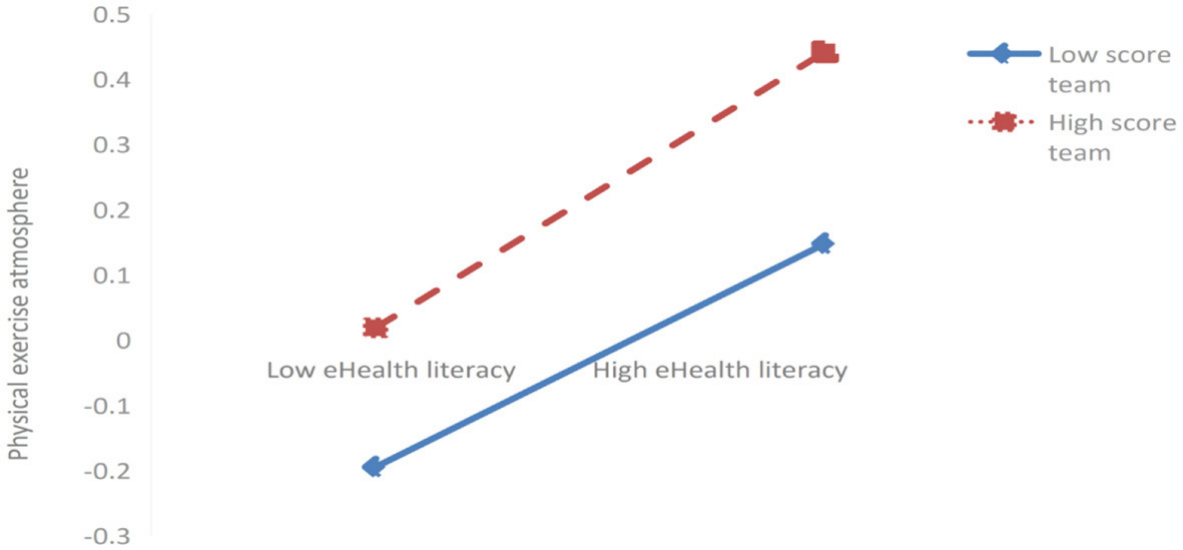
The mediating effect of eHealth literacy on the relationship between peer support and physical exercise atmosphere.

In the first half of mediating effect (as shown in Figure 5), for students with low scores on eHealth literacy (*M*-1SD), peer support exerted a significantly positive effect on physical exercise atmosphere (*simple slope* = 0.131, *t* = 3.381, *P* < 0.001). However, for students with high scores on eHealth literacy (*M*+1SD), peer support exerted a more significantly positive impact on physical exercise atmosphere (*simple slope* = 0.212, *t* = 5.473, *P* > 0.001). This indicates that eHealth literacy can significantly mediate the intensity of the impact of peer support on physical exercise atmosphere. Specifically, when the score on eHealth literacy increases, the positive impact of peer support on physical exercise atmosphere gradually increases. When the score on eHealth literacy decreases, the positive impact of peer support on physical exercise atmosphere shows a downward trend.

## 4 Discussion

### 4.1 The relationship between peer support and college students' sleep quality

This study revealed that peer support can exert a significantly negative impact on the sleep quality of college students. In other words, for college students, higher peer support level is associated with lower score of sleep quality and better sleep quality. This result is consistent with previous studies (Wu J. et al., 2023). Ecosystem theory suggests that individual development largely depends on their environment (Bronfenbrenner, 1986), and peer support is a microsystem environment that directly influences student development (Gloria et al., 2005). Considering this study, after students enter university, they break through the barriers of their inherent family and rely more on university resources to achieve independence and social development (Ross and Mirowsky, 2006). In such circumstances, family support gradually decreases, but students gain more peer support from increased companionship, mutual help, and emotional communication (Finkenauer and Righetti, 2011). This not only provides college students with more opportunities for emotional expression and regulation, but also fosters a greater sense of belonging and safety, leading to good sleep quality. Additionally, peer support creates a conducive environment for college students to acquire sports skills and enhance their social abilities (Wang Fu et al., 2018). It motivates them to engage in group activities like sports and club participation, thereby activating physiological and psychological mechanisms that can improve sleep quality. Generally speaking, when college students perceive more peer support, they are more likely to actively adjust their mental health status and consequently improve their sleep quality.

### 4.2 The moderating role of physical exercise atmosphere

This study revealed that physical exercise atmosphere plays a mediating effect between peer support and sleep quality. In other words, peer support can indirectly influence the sleep quality of college students through physical exercise atmosphere.

On the one hand, peer support can exert a significantly positive impact on physical exercise atmosphere (Wu J. T. et al., 2023). This is consistent with the results of previous studies. According to the theory of synergy, when external factors or group dynamics reach a critical threshold, a qualitative transformation occurs, giving rise to synergistic effects. These effects drive the system from disorder to order, generating stable structures from chaos (Ling, 2013). Strong peer support can cultivate a stable and harmonious interpersonal atmosphere for college students (Dong et al., 2013), facilitating positive exchanges of trust and emotional communication. This, in turn, fosters students' acceptance of exercise behavior, bolsters the exercise beliefs and enthusiasm of group members (Schaefer et al., 1981), and leads to collective efficacy through collaborative efforts among peers (Chen and Zhang, 2015). In short, with peer support, individuals' exercise beliefs, attitudes, and behaviors transition from disorder to order, thereby cultivating or perceiving an optimal physical exercise atmosphere.

On the other hand, physical exercise atmosphere can exert a significantly negative impact on college students' sleep quality. In other words, higher perceived exercise atmosphere level is associated with lower score of sleep quality and better sleep quality. This finding supports previous research views (Dong et al., 2023). According to the exercise persistence cognitive decision-making model (Chen et al., 2006), physical exercise atmosphere serves as an external force adjusting implicit psychology and promoting exercise persistence (Li et al., 2018). A positive exercise atmosphere can provide college students with a supportive environment for maintaining physical exercise behavior. The sense of belonging, exercise-related information, and peer relationships will subtly transform into emotional support, encouraging college students to continue participating in exercise (Xu and Dong, 2020), thus promoting their active engagement in physical activity. As college students increase the duration, intensity, and frequency of their physical exercise, it contributes to the development of a more comprehensive and systematic cognitive reappraisal mechanism and emotional regulation strategies. This, in turn, offers emotional support for enhancing their sleep quality. Moreover, elevating levels of physical activity assists individuals in improving vagus nerve function, stabilizing cortisol levels (Jensen et al., 2017), stimulating melatonin secretion from the pineal gland (Lian et al., 2021), and activating the hypothalamus to regulate the body's thermoregulation mechanism (Peng, 2008). These processes trigger and enhance slow-wave sleep, thereby boosting the sleep quality of college students.

### 4.3 The mediating effect of eHealth literacy

This research also discovered that eHealth literacy plays a role in moderating the relationships between peer support and sleep quality, physical exercise atmosphere and sleep quality, and peer support and physical exercise atmosphere. This partially validates the Knowledge—Attitude/Belief—Practice model. Moreover, the results indicated that the eHealth literacy level of college students serves as a protective factor influencing their psychology and behavior in the Internet era (Von Wagner et al., 2007). It can synergize with external environmental factors to positively regulate individual health behaviors.

On one hand, as the score of eHealth literacy increased, the positive influence of peer support on physical exercise atmosphere increased, while its negative impact on sleep quality became more apparent. This suggested that college students with higher eHealth literacy tend to have better sleep quality. Drawing from previous research, it's evident that health belief and health behavior can mutually influence each other among peers, leading to a collective effect of adopting healthy behaviors (Zhu, 2013). College students with high eHealth literacy levels often exhibit a strong willingness (Chen et al., 2022) to search for health information online (Lyu et al., 2024). During this search process, they might perceive and receive support from netizens and peers. This will accelerate their understanding of health management knowledge and help them build health belief, thus profoundly influencing their health decision-making and behaviors (Cui et al., 2021). When college students possess strong health beliefs and decision-making abilities, they might become more sensitive to positive atmospheres such as physical exercise and emotional regulation, and then become more proactive in engaging in healthy behaviors such as fitness exercise and leisure entertainment. In this process, college students not only perceive the leisure, entertainment, and physical exercise atmosphere constructed by peer support, but also engage in good emotional communication with peers, obtaining positive emotional experiences, and thereby improving their sleep quality.

On the other hand, when the score of eHealth literacy increased, the negative impact of physical exercise atmosphere on sleep quality became more prominent, indicating better sleep quality among college students. This aligns more closely with previous research results (Kim and Oh, 2021). According to the comprehensive eHealth use model (Bodie and Dutta, 2008), individuals with high e-health literacy tend to use Internet electronic products to address health problems, and promote their own health behavior by leveraging online health information. In this study, college students with higher levels of eHealth literacy tend to adopt positive health behaviors (Balay-Odao et al., 2021), actively participate in sports activities, and exercise more frequently (Wu and Jiang, 2022). This helps stimulate their awareness and perception of the surrounding sports environment, thereby enhancing their emotional experience and promoting their active integration into the physical exercise atmosphere. This positive exercise atmosphere helps promote emotional communication and enhance social skills among college students, thus enabling them to perceive physical exercise as a pleasurable activity for both body and mind, and as a means to fulfill their self-worth in society. This allows them to experience more respect and care, subsequently achieving stable psychological emotions and improving sleep quality as a result.

### 4.4 Research significance and limitations

By building a moderated mediation model to explore the relationships between peer support, physical exercise atmosphere, eHealth literacy and sleep quality, this study further verified the relationship between peer support and sleep quality and its influencing mechanism. Therefore, this study can offer valuable insights into improving the sleep quality of Chinese college students. Firstly, universities and colleges should prioritize enhancing students' interpersonal communication strategies and skills. This can be achieved through methods such as group counseling and practical teaching activities to strengthen their ability to coordinate interpersonal relationships. In turn, this can help them better receive acceptance, recognition, companionship, and support from their peers during their university years. Secondly, universities and colleges should fully implement a new integrated model of physical education teaching, emphasizing teaching, diligent training, and regular competitions. Additionally, they should organize sports competitions that have great appeal to students, aiming to create a positive and active physical exercise atmosphere. This will contribute to fostering a conducive environment for improving the sleep quality of college students. Thirdly, universities and colleges should promote eHealth knowledge through general education courses and activities like online health information retrieval competitions. Such efforts will continuously enhance college students' understanding of eHealth knowledge and learning, thereby improving their eHealth literacy and ultimately enhancing their sleep quality.

Admittedly, although this study preliminarily revealed the influencing mechanism of peer support, physical exercise atmosphere, eHealth literacy and sleep quality using a moderated mediation model, there are still some deficiencies that need to be further improved. (1) The research data was mainly collected through self-assessment questionnaires, which may have certain methodological effects. In future research, questionnaires should be collected through multiple channels. (2) As this study adopted a cross-sectional study design, it can only be used to explore the relationships between peer support, physical exercise atmosphere, eHealth literacy and sleep quality, and cannot make causal inference. In future research, experimental research or longitudinal design should be adopted. (3) In addition to the variables mentioned in this study, there must be other relevant factors that can affect the sleep quality of college students. Future research should make further exploration and reveal the influencing mechanism of sleep quality among college students more comprehensively. (4) Since the relevant information in this study was self-reported by Chinese college students, it may be influenced by personal biases and societal expectations. Future research could consider using a multi-agent evaluation method to mitigate any impact on the research results.

## 5 Conclusions

(1) Peer support can negatively affect college students' sleep quality. In other words, peer support can improve college students' sleep quality. (2) Physical exercise atmosphere plays a mediating role between peer support and sleep quality, that is, good peer support can improve college students' sleep quality through positive physical exercise atmosphere. (3) eHealth literacy can positively moderate the impact of peer support on physical exercise atmosphere, that is, the positive impact of peer support on the physical exercise atmosphere increases when eHealth literacy level increases. Additionally, eHealth literacy can negatively moderate the impact of peer support and physical exercise atmosphere on sleep quality. In other words, when eHealth literacy level increases, the negative impact of peer support and exercise atmosphere on sleep quality also increases accordingly, thereby improving the sleep quality of college students.

## Data availability statement

The raw data supporting the conclusions of this article will be made available by the author, without undue reservation.

## Ethics statement

The studies involving humans were approved by the Division of Science and Technology, Beijing Union University. The studies were conducted in accordance with the local legislation and institutional requirements. The participants provided their written informed consent to participate in this study. Written informed consent was obtained from the individual(s) for the publication of any potentially identifiable images or data included in this article.

## Author contributions

JL: Writing – review & editing, Writing – original draft, Visualization, Validation, Supervision, Software, Project administration, Methodology, Investigation, Formal analysis, Data curation, Conceptualization.
